# Opportunity, Challenge, or Both? Managing Adolescent Socioemotional and Mental Health During Web-Based Learning

**DOI:** 10.2196/26484

**Published:** 2021-09-15

**Authors:** Yolanda Evans, Jeffrey Hutchinson, Nusheen Ameenuddin

**Affiliations:** 1 Center for Child Health, Behavior and Development Division of Adolescent Medicine Seattle Children's Hospital Seattle, WA United States; 2 Wade Alliance Austin, TX United States; 3 Division of Community Pediatric and Adolescent Medicine Department of Pediatrics Mayo Clinic Rochester, MN United States

**Keywords:** pandemic, technology, media, bullying, mental health, distance learning

## Abstract

The transition to web-based learning during the COVID-19 pandemic has highlighted the need to consider the benefits of and the risks associated with web-based technology for education, media use, and access to resources. Prior to the pandemic, children and adolescents had in-person access to peers; social relationships; educators; health care providers; and, in some cases, mental health resources and medical care in schools and community settings. Due to the introduction of universal masking and physical distancing guidelines to prevent the spread of COVID-19 in early 2020, methods for accessing these resources have shifted dramatically, as people now rely on web-based platforms to access such resources. This viewpoint will explore equity in access to technology for web-based learning, mental health (with a focus on students of color), and the challenge of cultivating meaningful relationships on web-based platforms. Challenges and possible solutions will be offered.

## Equity in Web-Based Learning

Navigating physical isolation during the lockdown phases of the COVID-19 pandemic has created new challenges for individuals, particularly for children and adolescents who were required to rapidly adapt to distance learning by using technology to replace in-person schooling and daily social interactions. Prior to the pandemic, some students, such as those with serious or chronic medical conditions that require intermittent hospitalization, were homeschooled or had tutors as a regular part of their education. However, for the vast majority of school-age children in the United States, the transition to at-home web-based learning was abrupt. Due to the advent of the pandemic, old norms no longer exist. The pandemic has created a great disruption in more than education, as schools often provide meals; conduct various special education–based evaluations and therapies; facilitate physical activity and socialization; and offer career guidance, supervision, and sometimes even basic medical and mental care free of charge within their walls.

Although in-person schooling can be valuable for multiple children in multiple ways, not all schools are as well resourced as others. Among medical professionals and child advocates, there have been multiple concerns raised regarding the physical and mental health effects of in-person learning versus those of distance learning. Each side acknowledges child and community health but focuses on separate parts of the challenge (eg, prioritizing standard academics or prioritizing socioemotional learning) rather than seeing the whole picture and the impact that using technology for schooling has on children and families. The reopening of schools may have further exacerbated the risks of morbidity and mortality from COVID-19 in some communities, including immigrant communities and communities of color [1].

In a holistic approach to the technologic integration of learning in which health equity is the central goal, one should consider the following suggestions for addressing remote education:

- Educators, law makers, and medical institutions should incorporate feedback from the most affected communities, including parents and teachers of color in underresourced settings [1], in order to address inequities in education, including inequities in access to teachers, digital technology and equipment, and other services that were historically provided at school.
- Invest in infrastructure to provide reliable broadband internet to all students
- Create an equitable web-based learning experience for students who were previously excluded from full-time in-person learning due to medical conditions or other reasons

## Mental Health: Special Focus on Youth From Black, Indigenous, and Persons of Color Communities

In December 2019, a National Institute of Mental Health director’s message discussed alarming updates on Black youth suicide [2]. In the message, data from the Emergency Taskforce on Black Youth Suicide and Mental Health were discussed. Suicide rates among Black youth have been increasing [3]. In a group that has long faced inequities in access to mental health services and ongoing oppressive systems in school discipline, justice, and education, this seemingly resilient group of youths has started to exhibit increases in suicidality [4,5]. In 2020, continued trauma and stress have added up for Black youth and youth of color. This includes, but is not limited to, increased morbidity and mortality from COVID-19 among Americans in Black, Indigenous, and Persons of Color (BIPOC) communities [6]. In addition, unprecedented exposure through traditional and social media to the murders of Black citizens at the hands of law enforcement and the inhuman caging of immigrant children has taken a toll [7].

BIPOC youth, who are strong and resilient, are also hurting. The pandemic has magnified the inequities faced by all youth of color in the United States, and Black, Latino, and Indigenous communities are experiencing higher mortality rates [6,8] from infection, higher rates of unemployment, and disproportionate stressors related to housing and food insecurity [9,10]. Mental health and health care experts in Western Washington have also reported an increase in the number of youth of color expressing anxiety. Further, parents and caregivers are seeking advice on how to cope with stress themselves and how to guide their children through symptoms of anxiety, despair, and powerlessness.

Concurrent with the increased amount of stressors, the availability of mental health professionals has also been further strained. School closures have resulted in the loss of a key resource for mental health services that aim to help children and adolescents [11]. The number of licensed mental health providers with experience and expertise in working with youth from Black, Latino, and Indigenous communities was limited before the pandemic [4]. Now, families may face longer waiting times for mental health services, payment challenges (as providers may have switched to only accepting out-of-pocket payments), and limited in-person availability during the transition to telehealth services that promote safety during the pandemic.

In order to address the need for mental health support services, particularly those with a specific focus on BIPOC youth, one should consider the following possible solutions:

- Encourage patients and families to seek support from a health care provider for concerns about mental health
  Due to potential delays in access or longer-than-normal wait times, individuals should be encouraged to seek mental health resources early instead of waiting until symptoms affect their activities of daily living. 
- Providers should actively screen all patients for depression [12,13] and anxiety if they present to a clinical setting for nonmental health concerns.
- Education and health care institutions should ask about the mental health impacts of race-based violence that children witness on traditional and social media.

## Opportunities: Curating Web-Based Friendships

As previously stated, the COVID-19 pandemic has unfortunately resulted in multiple school closings in the United States since March 2020. This has had subsequent and variable consequences among different racial, ethnic, cultural, and socioeconomic groups. The American Academy of Pediatrics recognizes that essential development is best achieved in person and supports efforts for having children return to socializing and learning in the same space. To meet the future needs of students undergoing web-based learning, people must acknowledge that children are not monolithic and that the impacts of web-based learning are variable. The idealized model in which students are well resourced, are popular, and are involved in extracurricular activities ignores the students who were struggling in the social environment of school before the pandemic. The social pressure of school was relieved for some students who experienced regular bullying and microaggressions prior to the enforcement of web-based learning, while the high-achieving students experienced less social reinforcement and a lower sense of belonging [14]. However, for other students, the transition to web-based schooling resulted in the loss of their only safe place away from the troubles and trauma at home [15].

Although some of the negative impacts of social media can be unrelenting (eg, influences on body image, mood, and cyberbullying), there are children and teenagers who are discovering that they have the ability to conscientiously curate their friend groups and social experiences on web-based platforms. There are also youth finding their “online voice” through individual advocacy, like Mari Copeny, who continues to highlight the Flint, Michigan water crisis, and through group advocacy, like Future Coalition, a national network and community for youth-led organizations and youth leaders [16]. Current digital natives are learning how to mute, block, and unfriend people with expertise and engage with people on their terms. Prior to the pandemic, social media and digital platforms offered a sense of community, allowed for the exploration of identity, and offered a social forum for expanding friendships beyond physically proximate peers. During the pandemic, youth have relied on digital spaces to maintain and create social connections. These digital spaces provide parents and school-age children with the ability to make their web-based interactions positive, and future remote learning may be an option for families that require a safe or physically distanced environment.

One should consider the following suggestions to aid youth in building supportive friendships in web-based settings:

- Researchers can explore the incidence of students building supportive web-based relationships and their ability to avoid cyberbullying and negative peer influences.
- Adults in the education sphere can provide guidance to students on setting boundaries in web-based relationships. This includes avoiding hostile digital spaces, discontinuing social media profiles that induce stress and poor self-esteem, and reviewing aspects of positive peer relationships (eg, mutual respect, trust, empathy, and support) that can be sought in both web-based and in-person interactions.
- Medical providers can provide resources to families to help students build social skills for both web-based and in-person encounters that are applicable to encounters beyond the classroom [10].

## Conclusion

As the ongoing pandemic presents unprecedented challenges to pediatricians, mental health professionals, educators, and families, it is also important to remember that ingenuity can result in unique ways to cope, connect, and coordinate care. It is also important to make solid investments in infrastructure, such as access to broadband internet, mental health, and positive social connection, to minimize inequities. As care providers who care for children and adolescents, we must plan how to improve and sustain the education of youth and their socioemotional and mental health. We must also place specific focus on BIPOC communities, which have been disproportionately affected by the pandemic. Further, we must consider the widening of the digital divide as we develop methods for keeping children and adolescents engaged socially and educationally while considering the wider social impact on the hardest hit communities. The internet can be a great tool for supporting marginalized adolescents when it is available and is intentionally used. Physicians and educators should be prepared to equip families with the tools needed to explore the internet in healthy ways that open minds and promote self-studying and making connections [17]. The experiences and lessons gained from remote learning during the pandemic can be used to provide people with better learning outcomes as we face future challenges.

## Abbreviations

BIPOC: Black, Indigenous, and Persons of Color
